# A framework for the development of effective anti-metastatic agents

**DOI:** 10.1038/s41571-018-0134-8

**Published:** 2018-12-04

**Authors:** Robin L. Anderson, Theo Balasas, Juliana Callaghan, R. Charles Coombes, Jeff Evans, Jacqueline A. Hall, Sally Kinrade, David Jones, Paul S. Jones, Rob Jones, John F. Marshall, Maria Beatrice Panico, Jacqui A. Shaw, Patricia S. Steeg, Mark Sullivan, Warwick Tong, Andrew D. Westwell, James W. A. Ritchie, on behalf of the Cancer Research UK and Cancer Therapeutics CRC Australia Metastasis Working Group

**Affiliations:** 1 Translational Breast Cancer Program, Olivia Newton-John Cancer Research Institute, Heidelberg, Victoria Australia; 2 School of Cancer Medicine, La Trobe University, Bundoora, Victoria Australia; 3 Cancer Therapeutics Cooperative Research Centre (CTx), Melbourne, Victoria Australia; 4 Commercial Partnerships, Cancer Research UK (CRUK), London, UK; 5 Research and Innovation Services, University of Portsmouth, Portsmouth, Hampshire UK; 6 Department of Surgery and Cancer, Imperial College London, Hammersmith Hospital, London, UK; 7 Institute of Cancer Sciences, University of Glasgow, Glasgow, Scotland UK; 8 Research and Development, Vivacitv Ltd, Chesham, Buckinghamshire UK; 9 Medicines Development for Global Health, Southbank, Victoria Australia; 10 Medicines and Healthcare Products Regulatory Agency, London, UK; 11 Centre for Drug Development, CRUK, London, UK; 12 Queen Mary University of London, Barts Cancer Institute, London, UK; 13 Leicester Cancer Research Centre, University of Leicester, Leicester, Leicestershire UK; 14 Women’s Malignancies Branch, Center for Cancer Research, National Cancer Institute, Bethesda, MD USA; 15 School of Pharmacy and Pharmaceutical Sciences, Cardiff University, Cardiff, Wales UK

**Keywords:** Metastasis, Drug development, Drug therapy, Tumour biomarkers, Biomarkers

## Abstract

Most cancer-related deaths are a result of metastasis, and thus the importance of this process as a target of therapy cannot be understated. By asking ‘how can we effectively treat cancer?’, we do not capture the complexity of a disease encompassing >200 different cancer types — many consisting of multiple subtypes — with considerable intratumoural heterogeneity, which can result in variable responses to a specific therapy. Moreover, we have much less information on the pathophysiological characteristics of metastases than is available for the primary tumour. Most disseminated tumour cells that arrive in distant tissues, surrounded by unfamiliar cells and a foreign microenvironment, are likely to die; however, those that survive can generate metastatic tumours with a markedly different biology from that of the primary tumour. To treat metastasis effectively, we must inhibit fundamental metastatic processes and develop specific preclinical and clinical strategies that do not rely on primary tumour responses. To address this crucial issue, Cancer Research UK and Cancer Therapeutics CRC Australia formed a Metastasis Working Group with representatives from not-for-profit, academic, government, industry and regulatory bodies in order to develop recommendations on how to tackle the challenges associated with treating (micro)metastatic disease. Herein, we describe the challenges identified as well as the proposed approaches for discovering and developing anticancer agents designed specifically to prevent or delay the metastatic outgrowth of cancer.

## Abstract (web summary)

Most cancer-related deaths are attributable to metastasis, but few treatments are specifically designed to disrupt this process. In this Position Paper, representatives of the joint Cancer Research UK and Cancer Therapeutics CRC Australia Metastasis Working Group describe the challenges associated with discovering and developing anticancer agents designed specifically to prevent or delay the metastatic outgrowth of cancer and provide guidance on how these challenges might be overcome.

## Key points

- Metastasis is associated with a poor patient prognosis and is the foremost cause of cancer-related death, with approximately 90% of patients who succumb to cancer dying of metastatic disease.
- Metastasis is inherently complex, with different distant sites having a distinct and specific extracellular matrix and cellular composition compared with that of the originating site, and therefore metastases must be considered biologically different from the primary tumour.
- The standard cancer drug discovery and development pathway, including that for molecularly targeted and immunotherapies, generally ignores the ability of experimental medicines to inhibit metastasis.
- A wealth of potential preclinical targets for anti-metastatic drug discovery and development have already been identified but remain to be validated using appropriate preclinical models that reflect the pathogenesis of metastatic disease in patients.
- Despite some successes in the treatment of bone metastases, following extensive analyses in preclinical models, multiple late-stage failures in clinical development have resulted in anti-metastatic drug development efforts being deprioritized by the pharmaceutical industry.
- Successful development of effective anti-metastatic therapies will require the regulatory agencies to work together with researchers, drug developers and statisticians to redefine the clinical development paradigm in order to encourage development of this complex but high-potential category of oncology drugs.

## Introduction

Metastasis of cancer to distal sites is associated with poor patient prognosis and is the foremost cause of cancer-related death^1^, with approximately 90% of patients who succumb to cancer dying of metastatic disease^2^. Despite the advent of effective immunotherapies within the past decade, the majority of patients with advanced-stage and/or high-risk cancers continue to die as a direct result of metastatic disease or owing to complications of its treatment^3^. Indeed, improvements in the survival of patients with cancer over time have not equally benefited those with metastatic disease^4^.

Nevertheless, most standard-of-care treatments and new molecularly targeted therapies, including immunotherapies, were developed on the basis of initial evidence of anticancer activity — either direct or via immune system engagement — obtained in preclinical studies with tumorigenesis and/or primary growth, not metastatic activity, as the main end points. Similarly, clinical drug development generally relies on the demonstration of tumour shrinkage according to the radiological Response Evaluation Criteria for Solid Tumors (RECIST)^5,6^, with confirmatory improvements in clinical outcomes, ignoring the ability to inhibit metastasis. Only after clinically meaningful tumour responses and/or improvements in patient survival have been demonstrated in the metastatic setting will the drug be tested in adjuvant trials, with the aim of preventing or delaying the development of overt metastatic disease. Consequently, a paucity of preclinical discovery and thus clinical development exists for agents targeting the biological mechanisms underlying the metastatic process.

An urgent need remains for novel therapeutic strategies and agents that prevent the establishment of and/or tissue colonization by metastases, which ultimately lead to organ failure, morbidity and death. In addition, new strategies must be developed to facilitate clinical testing of therapeutic agents with an anti-metastatic mechanism of action. To assist the cancer drug discovery and development community in addressing this critical issue, Cancer Research UK (CRUK), Cancer Research Technology (CRT) and Cancer Therapeutics CRC Australia (CTx) formed a Metastasis Working Group with representatives from academia, industry, government and regulatory bodies in order to develop recommendations on how to surmount the challenges associated with treating metastatic disease. This article provides an overview of these challenges and describes the Metastasis Working Group recommendations on best practices for the discovery and development of anticancer agents designed specifically to circumvent metastasis, with consideration given to their implementation in clinical trials.

## Challenges

The development of new effective medicines that interrupt the primary causes of metastasis is a daunting but important challenge. Mechanistically, metastatic tumour cells are genetically unstable, and in most cancers no single dominant pathway is likely to control metastasis^7^. Indeed, the signalling pathways driving metastasis can vary between primary and secondary tumours and between metastases that arise at different sites^8^. The target of translational research efforts is often an occult population of tumour cells disseminating from the primary tumour, sitting dormant in a sanctuary site or constituting a micrometastasis in a distant organ. Selection for clinical testing of candidate anti-metastatic agents that might have limited or no effect on conventional preclinical outcomes, such as primary tumour growth, is another key challenge. In addition, validated biomarkers that can be used to increase the efficiency of mechanistic experiments and accelerate drug development are rarely available.

**Fig. 1:**
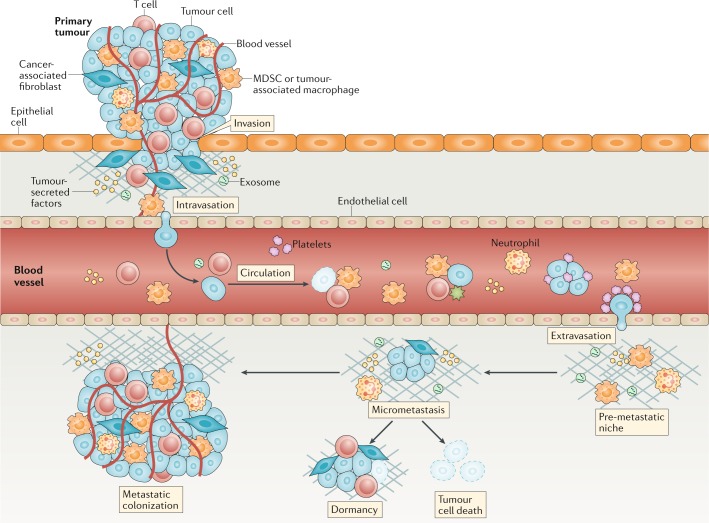
Overview of metastasis. Metastasis is a complex multistep process, and the very concept of designing a metastasis-specific therapeutic must consider which part of the process is best to target. Given that metastases are derived mainly from invasive tumours, therapeutic efforts have often targeted the intrinsic invasive propensity of tumour cells^150,151^. Tumour cell production of angiogenic factors and TGFβ can activate endothelial cells and fibroblasts to remodel tissues and promote tumour cell invasion of stromal-modified spaces^152^. Targeting stromal elements in cancers remains an active area of research^153–157^. Intravasation of tumour cells is promoted by binding to macrophages that cause transient permeability in the vasculature^158^; thus, targeting tumour-associated macrophages might reduce the number of circulating tumour cells (CTCs)^159^. Multiple factors intrinsic to tumour cells (including epithelial-to-mesenchymal transition, production of proteases and migratory capacity) improve intravasation, often via effects on cell types including fibroblasts, neutrophils and macrophages^160^. Most tumour cells that enter the vasculature die as a result of hydrodynamic physical damage or leukocyte attack^160^. However, platelets can bind to and protect CTCs and improve their ability to establish secondary sites^161^. Platelet–CTC aggregates settled at distant sites can release cytokines that attract granulocytes^162^; targeting platelets or granulocyte recruitment can prevent metastasis^162^. Additionally, abrogation of platelet–CTC binding, leading to a reduction in the number of circulating and potentially metastatic cells^163^, might explain the suppression of metastasis by aspirin in breast and prostate cancer models^164^. Survival and proliferation of newly deposited cancer cells in a metastatic site are arguably the most important stages of the metastatic process. Cancers with a propensity to metastasize do not grow in all organs, indicating that a limited number of organs provide a suitable stromal environment for their colonization. Preferred colonization sites, termed pre-metastatic niches, can be prepared in advance of the arrival of disseminated tumour cells through the actions of myeloid-derived suppressor cells (MDSCs) and tumour cell-derived extracellular vesicles (EVs), such as exosomes^17,165^. Whether this process can provide novel therapeutic targets to limit the arrest and survival of metastatic cells remains unclear, with development of EV-specific drugs, for example, creating a challenge^166^. Evidence also supports roles for neutrophils^167,168^ and MDSCs^169^ in metastatic colonization. Evasion of the antitumour immune response is another critical factor in metastatic colonization. No single tumour type seems to exhibit all these mechanisms; therefore, targeting any one stage of the metastatic process requires a tumour-specific understanding of the mechanisms involved.

Across all cancers, the extent to which tumour cells have left the primary tumour and established occult (micro)metastases before patient diagnosis is acknowledged to be poorly characterized^9,10^. Tumour cells might become invasive early in cancer development, and thus prevention of dissemination from a primary tumour is unlikely to be a clinically successful strategy owing to the presence of pre-existing but undetected metastases; however, prevention of secondary metastases is a plausible rationale for intervention. Metastatic dissemination is a multistage process, and various points of intervention have been identified and credentialed at the preclinical level (Fig. 1). These include targeting the initial steps of invasion and migration away from the primary tumour, entry into the circulation (intravasation) and extravasation at a distant site. As with invasion, traversal of the circulatory system might also be an early event in cancer development, leading to the proposal that drug development efforts should also take into consideration the abrogation of metastatic colonization — that is, the outgrowth of a lesion in a foreign environment. Several aspects of metastatic colonization are distinct from primary tumour formation and could influence drug development. Moreover, multiple studies have revealed that tumour cells colonizing distant organs can differ from those of the primary tumour in many respects^10–13^, revealing potential therapeutic targets. Changes in tissue microenvironments that facilitate metastatic colonization are incompletely characterized and might begin before the arrival of metastatic tumour cells^14,15^. Several approaches for targeting such secondary sites have been proposed. Intervening early to disrupt the ‘pre-metastatic niche’ is one potential strategy, while therapy to either maintain dormancy or induce the death of cells in micrometastatic lesions is another. Whereas metastases that are detected before commencement of first-line therapy can sometimes be treated using radiation therapy, new and less deleterious therapies that are additive or synergistic with the standards of care are a priority for the treatment of occult metastatic disease.

The metastatic cell is a single genetic entity derived from a mass of cells possessing extensive genetic heterogeneity and consequent plasticity^8^. The distant tissue site will also have a distinct and specific extracellular matrix and cellular composition compared with that of the tumour tissue from which the metastatic cell originated. Metastases must therefore be considered biologically different from primary tumour cells, at least in the early stages of outgrowth. Studies specifically designed to examine the biological development of metastases are not common, and if we hope to target metastases effectively, we must gain a more complete understanding of the underlying biology. This issue is complex, although some successes have already been achieved, for example, in understanding the processes of pancreatic cancer metastasis^16,17^.

**Fig. 2:**
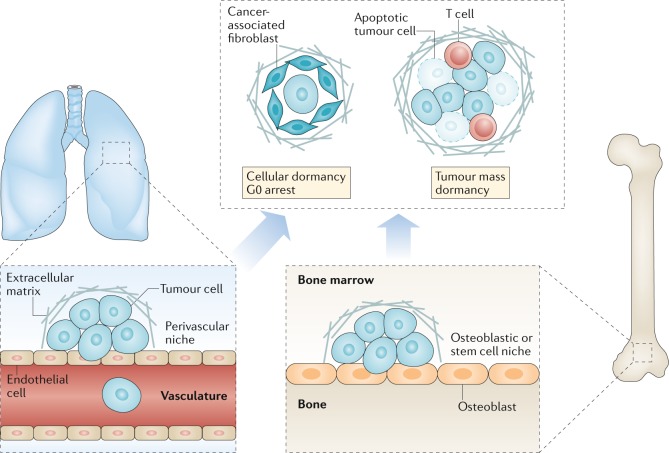
Dormancy and the metastatic niche. Metastatic latency is more pronounced for certain types of cancer, notably breast and prostate cancer and melanoma^170^. The detection of disseminated tumour cells (DTCs) in bone marrow aspirates, obtained long after eradication of the primary tumour, verifies the presence of dormant tumour cells and is predictive of disease recurrence^171–173^. Dormant tumour cells are proposed to exist either as single cells in a state of cell cycle arrest or as small masses of cells that fail to expand into clinically detectable lesions, possibly owing to failure of angiogenesis, balanced rates of proliferation and apoptosis or effective immunosurveillance^174^. Cells derived from these clusters that are too small to be detected by normal clinical imaging are presumably the source of circulating tumour cells detected in some patients after successful treatment of the primary tumour. The metastatic niche is likely to vary between different organs, reflecting the tissue-specific nature of the microenvironment in which the DTC is located; extensive crosstalk occurs between the tumour cells, stromal cells and extracellular matrix components of the niche. Various niches have been proposed, including the perivascular niche associated with the vasculature^22^, the haematopoietic stem cell niche of the bone marrow^175^ and the osteoblastic niche in bone^176^. The factors that maintain tumour cell dormancy in these niches are starting to be unravelled, with different extracellular matrix components, cytokines and other proteins being implicated for different cancer types and niches. Far less is understood about how dormancy is broken, which could occur following failure of immunosurveillance, in response to inflammation triggered by trauma or an infection or perhaps as a result of ageing-related deficiencies in tissue homeostasis. With regard to therapy, the challenge is to decide whether the aim should be to retain the DTCs in a dormant state or, instead, to disrupt their niche and dormancy, thereby rendering them susceptible to apoptotic or anoikic death and/or to chemotherapy.

Metastatic dormancy is defined by an unusually long disease-free interval (months, years or even decades depending on the cancer type) between removal or successful therapy of the primary tumour and subsequent clinical relapse with disseminated disease. Metastatic dormancy can be achieved by many means. Tumour cells can exit from the cell cycle or balance their proliferation and apoptosis. Host cells can limit angiogenesis or alter anticancer immune responses, resulting in immunoediting (with an equilibrium between immune elimination and escape of tumour cells)^18^. Tumour cell dormancy also alters chemotherapeutic efficacy, either because the non-dividing cells are more resistant to such treatment^19^ or because they are protected by cellular and extracellular components of their microenvironment^20–22^ (Fig. 2).

The optimal means of selecting patients with a predictable risk and rate of disease progression for enrolment in a clinical trial of an anti-metastatic agent remain largely unknown. This challenge is compounded by the inability to reliably quantify the prevalence and extent of occult metastatic disease at enrolment. Because adjuvant clinical trials are often conducted only after positive results have been obtained in early phase clinical studies involving patients with advanced-stage (metastatic) disease and require considerable funding, large numbers of patients and long follow-up durations to capture the primary outcome measure (for example, disease-free survival (DFS)), new non-conventional clinical development approaches are needed. The clinical trial design might vary depending on the patient population, the dosing schema (maximum tolerated versus biologically effective dose, limited cycles versus maintenance therapy and scheduling in relation to standard-of-care therapy), the primary end points and the available biomarkers of activity. In most cases, such trials will also need to be initiated without a history of regulatory approval of the experimental agent.

## Missteps and a home run

Several past efforts to target the mechanisms of tumour metastasis have resulted in failure. Matrix metalloproteinase (MMP) inhibitors were developed as anti-metastatic agents on the basis of their capacity to inhibit tissue invasion by tumour cells early in the metastatic cascade and their cytostatic activity in preclinical models^23,24^. Subsequent studies enabled a more nuanced understanding of the metastasis-promoting as well as the metastasis-limiting effects of the MMPs and revealed that several other proteases have overlapping functions^25^. The preclinical models used had limited relevance to the clinical testing scenario, with the experimental design typically involving initiation of MMP inhibitor treatment soon after tumour cell inoculation, to form either a primary tumour or experimental metastases. In this setting, the MMP inhibitors were shown to be effective^26^; however, in the clinic, MMP inhibitors were tested in patients with advanced-stage disease, usually with drug-resistant metastatic disease. As a result, these phase II and phase III clinical trials of MMP inhibitors failed to show strong signals of efficacy^27,28^. In addition, the early drugs had broad spectrum anti-protease activity and were characterized by serious adverse effects in patients^26^. Other examples of anti-metastatic agents for which promising preclinical activity has not been translated into clinical efficacy include cilengitide (targeting αvβ3 and αvβ5 integrins on angiogenic blood vessels)^29^, and dasatinib and saracatinib (targeting SRC and BCR–ABL1)^4^. These late-stage failures in clinical development have resulted in major financial losses, leading to anti-metastatic drug development being deprioritized by the pharmaceutical industry.

Conversely, successes have been achieved in the treatment of bone metastases, following extensive analyses in preclinical models, either with antibodies targeting receptor activator of NF-κB ligand (RANKL; also known as TNFSF11) or with a class of drugs known as bisphosphonates. Both of these therapies interrupt the ‘vicious cycle’ of bone metastasis. The bone metastatic vicious cycle is a specific example of tumour cell–microenvironment interactions that are likely to occur at other metastatic sites (albeit via differing mechanisms). In bone, tumour cells produce factors that activate osteoblasts to produce RANKL, which activates osteoclasts that subsequently degrade bone, releasing a host of growth factors that stimulate metastatic colonization by tumour cells^30^. Denosumab is a humanized monoclonal antibody targeting RANKL that has been shown in preclinical experiments to bind with its target in the bones of healthy transgenic mice expressing chimeric mouse–human RANKL^31^. In initial clinical trials in the metastatic setting, traditional tumour growth inhibition or survival end points were not used as outcome measures with this cytostatic agent; instead, a reduction in skeletal-related events (SREs) was the primary end point^32,33^. Deleterious SREs, such as a bone fracture from expansion of an existing metastasis or a new metastasis, were essentially direct readouts of the extent of metastatic burden. Accordingly, patients with breast or prostate cancers were enrolled because they are prone to developing bone metastases. Significant reductions in the incidence of SREs in patients treated with denosumab compared with those who received standard care were observed for both types of cancer^34,35^. In men with bone-metastatic castration-resistant prostate cancer (CRPC), the median time to first on-study SRE with denosumab was 20.7 months versus 17.1 months with the bisphosphonate zoledronic acid (HR 0.82, 95% CI 0.71–0.95; *P* = 0.0002 for non-inferiority; *P* = 0.008 for superiority)^34^. In women with advanced-stage breast cancer, denosumab was superior to zoledronic acid in delaying the time to first on-study SRE (HR 0.82, 95% CI 0.71–0.95; *P* = 0.01) and also the time to first and subsequent on-study SREs (rate ratio 0.77, 95% CI 0.66–0.89; *P* = 0.001)^35^. Denosumab was then compared with placebo in adjuvant trials and delayed initial bone metastasis in patients with CRPC by a median of 4.2 months^36^. However, in placebo-controlled trials involving patients with breast cancer, adjuvant denosumab was associated with only a minor^37^ or no^38^ reduction in the DFS or overall survival (OS). The reasons for this disparity between SREs and OS outcomes are not entirely clear but might reflect a shift in bone metabolism that reduces the incidence of SREs without direct effects on cancer progression.

The value of bisphosphonates in reducing bone resorption and bone metastasis through direct targeting of osteoclasts has been demonstrated in many preclinical studies^39^. In addition, bisphosphonates have broader anticancer activity against metastatic lesions in visceral organs, possibly through inhibition of angiogenesis or through inhibition of M2-like macrophages in other tissues^39,40^. A meta-analysis of clinical trials using bisphosphonates, supported by earlier preclinical data, has demonstrated reduced bone metastases and prolonged OS, at least in postmenopausal women with early stage breast cancer^41^. No benefit of bisphosphonate treatment was observed for premenopausal women^41^.

## Preclinical drug development

### Target identification

Many targets with known or proposed roles in metastasis could be candidates for drug development (Table 1). These potential targets have been identified on the basis of associations between gene targets (often mutated) and metastasis or poor survival in patients^42,43^, targeted manipulation of genes to alter metastasis in preclinical models^44–46^, functional genomic screens^47–49^ and drug repurposing efforts^50^. In addition to functional preclinical experiments, evidence that the target is associated with metastasis in the human disease is required. For the development of a targeted therapy, such as a small molecule, a peptide or an antibody, knowledge of the biological activity of the target is essential for supporting development of a functional biomarker. Targets with strong correlations with metastasis but not passing the functional tests might serve as biomarkers of a response.

**Table 1:** Selected preclinical data for potential anti-metastatic therapies

Agent	Target	Preclinical data^a^
***Antibodies***		
Anti-CCL2	CCL2 (chemokine)	Prevented mobilization of myeloid cells from the bone marrow to colorectal liver metastases and thereby reduced metastasis^177^
Anti-BMP6	BMP6 (TGFβ superfamily cytokine)	Reduced osteoblastic bone metastasis from prostate cancer^178^
Anti-PTHrP	PTHrP (hormone involved in bone vicious cycle)	Reduced liver and bone metastasis of melanoma^179^
Anti-N-cadherin	N-cadherin (mesenchymal cadherin)	Reduced prostate cancer muscle invasion and induced tumour cell apoptosis^180^
Anti-CD24	CD24 (GPI-linked sialo-glycoprotein)	Reduced lung metastasis of bladder cancer^181^
Anti-CDCP1	Protease cleavage site of CDCP1	Prevented lung metastasis by inducing poly(ADP-ribose) polymerase-mediated cell death^182^
Anti-TSPAN8	TSPAN8 (tumour-associated tetraspanin)	Reduced metastasis of epithelial ovarian cancer^183^
Anti-MT1-MMP	Membrane type 1 MMP	Reduced lung metastasis of melanoma^184^
***Small-molecule inhibitors***		
BL5923	CCR1 (CCL9 and/or CCL15 chemokine signalling)	Inhibited liver metastasis of CRC by blocking recruitment of myeloid cells^185^
SD208	TGFβ receptor 1	Reduced melanoma and prostate bone metastasis and decreased progression of established lesions^186,187^
CCT129254	Multiple kinases (including ROCK, PI3K and AKT)	Inhibited melanoma lung metastasis^188^
Zibotentan	Endothelin 1	Prevented lung colonization by bladder cancer cells but had no effect on established metastases^189^
Debio 0719	Lysophosphatidic acid receptor 1 (fibrosis)	Decreased lung and liver metastasis in breast cancer and induced tumour cell dormancy^11^
β-Aminopropionitrile	Lysyl oxidases	Prevented breast cancer metastasis but had no effect on existing lesions^190^
CCT365623	Lysyl oxidases	Prevented metastasis of breast cancer^191^
CA-074	Cathepsin B inhibitor	Prevention of bone metastasis and shrinkage of existing bone metastases in a breast cancer model^192^
Napabucasin	Unclear (STAT3 and cancer stem cell pathways)	Reduced metastasis of pancreatic and colon cancers^193^
HO-3867	Unclear (STAT3 signalling and reversion of mutant p53 to a wild-type phenotype)	Reduced metastasis of ovarian cancer^194^
IRAK inhibitor and ginsenosides	IRAK1	Reversed paclitaxel resistance and reduced metastasis of TNBC^195^
Bafetinib	LYN and BCR–ABL1	Decreased liver metastasis in a breast cancer model^196^
KPT-6566	PIN1 (prolyl isomerase that regulates proline-directed kinase signalling)	Decreased lung metastasis of TNBC^197^
SF2523	Dual PI3K and BRD4 inhibitor (MYC-mediating factors)	Reduced regional colonic lymph node metastasis and shrank established metastases in pancreatic carcinoma model^198^
Nifuroxazide	Unclear (STAT3 signalling)	Inhibited lung and abdomen metastasis of CRC and shrank existing metastases^199^
AECHL-1 (triterpenoid)	Unclear (alters cytoskeletal dynamics and inhibits NF-κB-mediated MAPK activity)	Decreased lung metastasis of TNBC^200^
CCG-203971	Unclear (inhibits the RHO–MRTF–SRF pathway)	Decreased lung metastasis in melanoma model^201^
Regorafenib	Multiple kinases (including angiogenic receptor tyrosine kinases)	Decreased lung metastasis of CRC (via activation of the protein tyrosine phosphatase SHP1) and shrank existing metastases^202^
GW3965	Liver X receptors	Inhibited brain metastasis of melanoma and shrank existing lesions^203^
Low-dose paclitaxel	Tubulin	Decreased lung metastasis of cholangiocarcinoma (via reduced nuclear import of the calcium-binding protein S100A4)^204^
Selumetinib	MEK	Decreased lung metastasis of TNBC^205^
G2	Fascin (actin-bundling protein)	Decreased lung metastases of breast cancer^206^
Zileuton	Arachidonate 5-lipoxygenase	Reduced spontaneous metastasis of MMTV-PyMT cells^168^
***Peptides***		
Bone metastasis-targeting peptide 78	Endoplasmic reticulum chaperone BiP	Reduced outgrowth of established lung and bone micrometastases in an advanced-stage breast cancer model^207^
T22	CXCR4 (SDF1 chemokine signalling)	Showed synergy with anti-CTLA-4 therapy in reducing the size of established melanoma metastases^208^
Ac-PhScN-NH_2_	α5β1 integrin (fibronectin receptor)	Inhibited bone metastasis, disease progression and lung colonization in a breast cancer model and shrank established lesions^209^
***Immunotherapies***		
*MTDH* DNA vaccine	MTDH	Induced T cell responses and prevented lung metastasis in a breast cancer model^210^
*LMP1* DNA vaccine	LMP1 viral antigen	Inhibited TC-1 lung metastasis in vivo via targeting of EBV LMPs^211^
***Others***		
Retinoic acid	Retinoic acid receptor (inhibits cell adhesion)	Inhibited melanoma lung metastasis by inhibiting tumour cell adhesion to the vascular endothelium and subendothelium^212^
IGF trap	IGF1R	Promoted apoptosis of colon and lung cancer cells in nascent liver metastases^213^
Ad.dcn (decorin-expressing oncolytic adenovirus)	Various (results in downregulation of MET, β-catenin and VEGFA)	Systemic delivery shrank established bone metastases of prostate cancer^214^
Cellax-DTX polymer (docetaxel–acetylated carboxymethylcellulose–PEG conjugate nanoparticles)	Tubulin (results in selective depletion of activated, cancer-associated fibroblasts)	Decreased development of pancreatic metastases^215^
*N*-acetylcysteine	Reactive oxygen species (antioxidant)	Inhibited liver metastasis of pancreatic cancer^216^
NM-NP-CFZ (neutrophil-mimicking-nanoparticles containing carfilzomib)	Inflammatory neutrophils	Prevented early lung metastases and shrank established metastases in mammary carcinoma models^217^

CDCP1, CUB domain-containing protein 1; CRC, colorectal cancer; CTLA-4, cytotoxic T lymphocyte antigen 4; EBV, Epstein–Barr virus; GPI, glycosylphosphatidylinositol; LMP, latent membrane protein; MMP, matrix metalloproteinase; MRTF, myocardin-related transcription factor; MTDH, metadherin (also known as LYRIC); PEG, polyethylene glycol; SRF, serum response factor; TNBC, triple-negative breast cancer. ^a^Unless otherwise noted, the intervention was shown to prevent or delay the development of metastasis.

### Preclinical modelling

Effective candidate identification relies on preclinical models that accurately recapitulate the disease pathogenesis in patients and, specifically, the particular process being targeted (that is, initial invasion, extravasation, the development of nascent metastases or metastatic outgrowth). Many commonly used preclinical tumour models are better suited for testing agents that have a direct antitumour effect, often on primary tumours, rather than clinically relevant effects on metastasis^51,52^. For numerous cancer types, the primary lesion can be well controlled by standard therapies (surgery, radiotherapy and/or chemotherapy) — the challenge is to control the onset and growth of secondary lesions.

No single preclinical model exists that wholly reflects metastasis in patients with cancer. Several different preclinical models of the type of cancer under study should be used when testing the anti-metastatic activity of a new drug to account for the diversity of the disease in patients^52^. If a molecularly targeted therapy is to be tested, an ideal preclinical model is one in which the target molecule promotes one or more steps in the metastatic process within that preclinical model. For example, inducible expression of the transcription factor Twist-related protein 1 in mice can drive epithelial-to-mesenchymal transition and result in increased metastasis of squamous cell carcinoma^53^. Other examples include prevention of metastases through targeting of SRC expression in orthotopic mouse models of human pancreatic adenocarcinoma^54^ or dual pharmacological inhibition of MET and VEGFR2 (ref.^55^) in various tumour models and tumour cell lines, or transgenic mice in which mammary tumour development and metastasis are driven by the expression of HER2 (ref.^56^) or E545K-mutant PIK3CA^57^. Examining drug efficacy in models with complex genetics, including in both metastatic driver and passenger pathways, can also be informative by reflecting the genomic complexity of patient tumours.

The fact that host tissues dictate the extent to which a tumour can metastasize is becoming increasingly evident. The tumour microenvironment is complex, varies extensively in different organs and is influenced by tumour–host cell interactions, physical and metabolic changes, and secreted cytokines, chemokines and growth factors^58^. As well as influencing tumour growth directly, these changes alter the capacity of the immune system to recognize and attack tumour cells^59^. These factors must be taken into account when selecting preclinical models in order to ensure that interventions are effective for the tumour type and/or organ of metastatic involvement^58^. For example, in a mouse model of prostate cancer, androgen ablation (by castration) was demonstrated to result in bone loss and enhanced the growth of disseminated tumour cells (DTCs) in the bone; however, detrimental exacerbation of bone metastasis could be overcome by administering bisphosphonates together with androgen ablation^60^.

No in vitro test adequately models the entire metastatic process; therefore, in vivo modelling is essential. Generally, metastasis models consist of either mouse tumours in a syngeneic host, thereby allowing for full engagement of the immune system, or human tumours engrafted into immunosuppressed hosts^52,61^. Mammals other than mice, such as rats^62^, are used occasionally, as are non-mammalian hosts such as zebrafish^63^, *Drosophila*^64^ and the chick embryo chorioallantoic membrane^65^.

Genetically engineered mouse models (GEMMs) use mice bearing oncogenes that initiate a primary tumour (quite often, multiple primary tumours), which in some cases progress to metastatic disease (for example, the LSL-*Kras*^G12D/+^; LSL-*Tp53*^R172H/+^; *Pdx1*-Cre KPC GEMM used for preclinical studies of both pancreatic cancer prevention and therapy^66^). When murine transplantable tumour models are used, cells can be introduced, preferably at orthotopic sites, to initiate primary tumour growth and subsequent spontaneous metastasis. A major advantage of such mouse metastasis models is the presence of matched stromal tissues that can recapitulate growth factor signalling and anticancer immune responses. Given the obvious and critical role of the host immune system in regulating metastasis^58,59,67^, models of murine tumours in immunocompetent syngeneic hosts should be included in the preclinical testing of any new therapeutic whenever possible.

Metastasis assays can also involve established cancer cell lines or tissues recovered directly from patients undergoing tumour biopsy sampling or resection. As opposed to a cell line that has drifted genomically during long-term culturing, patient-derived xenografts (PDXs) have the important advantage of more closely reflecting the genomic profile of the original tumour. PDXs are also reported to metastasize to the same organs as metastases in the donor patient^68–70^. However, xenograft models obviously lack competent immune regulation (owing to the need to avoid immune-mediated destruction of the transplanted allogeneic tumour cells). The advent of even more severely immunocompromised mice, such as non-obese diabetic–severe combined immunodeficient (NOD–SCID) and NOD–SCID–IL-2-receptor γ-chain-mutant (NSG) mice, has enabled a higher proportion of tumours to be established in such models, with more circulating tumour cells (CTCs) and metastases. However, in patients, tumours develop despite the presence of a competent immune system by evolving mechanisms to escape immune detection and/or destruction; therefore, various methods of incorporating a human immune system into these severely immunocompromised mice are the subject of active research^61^. CTCs isolated from 10 ml of blood have also been used to generate patient-derived CTC xenograft models (CDXs) and have several advantages over PDX models generated using tumour biopsy samples. These advantages include the development of tumours with a molecular profile generally similar to that of the primary tumour and single CDXs that respond to chemotherapy in the same way as the donor patient’s tumour; the ability to generate models for patients with tumours that are not amenable to biopsy sampling or surgery; and the use of a population of tumour cells that has already gained an invasive behaviour, reflecting intra-patient heterogeneity, and that can be used as a surrogate to study metastasis (reviewed in ref.^71^). Nevertheless, CDXs do have drawbacks, such as lack of a functional immune system, and can be challenging to establish for some tumour types.

Cell lines and dissociated murine tumours can also be injected haematogenously, intraperitoneally, intrasplenically or by other routes to circumvent primary tumour formation. This scenario might be justified for types of cancer in which initial seeding of tumour cells at distant sites commonly occurs before diagnosis. Such ‘experimental metastasis’ models offer the advantages of rapid metastatic progression and greater numbers of tumours, which can accelerate study throughput. However, these models might fail to recapitulate the aforementioned capacity of the primary tumour to fashion the pre-metastatic niche by releasing growth factors, cytokines, proteases and extracellular vesicles^14,72,73^ (Fig. 2).

Models with different sites of spontaneous metastatic dissemination that reflect the patterns of metastasis in patients are a further necessity for preclinical testing. The 4T1.2 mammary cancer model is an example of a mouse model that recapitulates the pattern of metastatic spread of its human counterpart, with the primary tumour generating spontaneous metastases in the lymph nodes, lungs and bones — major sites of metastasis in patients with breast cancer^45^. Clearly, therapies will need to be tailored for metastatic lesions in different organs because the microenvironments of bone, liver and brain, for example, are very different and therefore confer different properties to the tumour cells that successfully colonize these organs. For example, treatment with the RANKL antagonist osteoprotegerin effectively controls bone metastases, but not metastasis to the lungs in a mouse model of metastatic breast cancer^74^.

In general, too few preclinical metastasis models are available to adequately replicate the substantial heterogeneity of metastases in patients. Ideal models should be orthotopic, immunocompetent and able to produce metastases within a few months. Several common types of cancer, including melanoma, breast cancer and prostate cancer, can have a long latency — up to two decades between the initial treatment of the primary tumour and the development of distant metastases. For these cancers, evidence exists for very early dissemination of the tumour cells, even before diagnosis, followed by long periods of dormancy after extravasation into other tissues^22,75–80^ (Fig. 2). These findings and those of other similar studies emphasize the importance of developing therapies that can prevent the outgrowth of DTCs or that result in the death of very slowly cycling cells. Preclinical models that mimic this metastatic dormancy phenotype need to be developed to facilitate testing of such therapies and should incorporate current adjuvant treatments, such as hormone therapy for breast or prostate cancer. Current models involve the use of transplantable tumour cell lines with much delayed development of metastatic disease that becomes evident only after an extended time following successful resection of the primary tumour. Examples include the mammary tumour lines D2.OR^81^ and D2.A1-GFP^82^ and HEp3 cells isolated from the lymph node of a patient with head and neck squamous cell carcinoma^83^. Thus, various important features need to be considered when developing preclinical models of metastasis suitable to evaluation of experimental anti-metastatic agents (Box 1).

Box 1 Validating a potential anti-metastatic agent using preclinical in vivo models Animal models are required to provide mechanistic insights into the effects of experimental agents on the entire metastatic process and are conducted primarily in mice. Multiple factors are important to consider when establishing the experimental design if a translational goal is anticipated.

**Prevention of metastasis versus shrinkage of existing lesion**

Most preclinical metastasis experiments are focused on preventing the initial formation of a metastasis — tumour cells are injected into mice, and the experimental agent is delivered soon after and continuously, with the number and sizes of metastases quantified at the end point. The findings of such studies are more relevant to adjuvant trial designs given the close alignment of aims between the clinical setting and these models — that is, avoiding relapse owing to undetectable disease. Even so, metastatic tumours can be allowed to grow to reflect a more advanced-stage cancer setting.

**Spontaneous versus experimental metastasis**

Metastasis models in which neoplastic cells form a primary tumour and subsequently metastasize are the gold standard, but the low number of lesions produced over long periods of time is an important limitation; few such models exist. Other experimental models use haematogenous or other routes of tumour cell delivery and enable interrogation of the final stage of metastasis: metastatic colonization.

**The source of tumour cells**

Cell lines are easier to use, but long-term culture can result in cell lines that do not accurately recapitulate the actual clinical disease biology. Genetically engineered models generally have low frequencies of metastases and might not reproduce the genomic diversity of human tumours. By contrast, patient-derived xenografts and spheroids have been reported to closely replicate the tumour heterogeneity and evolution and the clinical course^70,149^. Ultimately, the use of multiple models is always preferable.

**The animal (typically mice)**

Given the increasing importance of immunotherapy, and the immune contributions to responses to standard therapies, use of syngeneic, immune-proficient mice is advisable. Indeed, multiple aspects of the microenvironment are contributors to the metastatic cascade and ideally should be considered in preclinical models.

**Site of injection**

Subcutaneous models should be avoided. For models of spontaneous metastasis, orthotopic injection of tumour cells is mandatory.

**Site of metastasis**

The sites of metastasis in the mouse model should reflect the characteristic sites of dissemination associated with the human disease being studied.

**Prior chemotherapy or radiation therapy, or concurrent therapy**

Any new therapeutic must be given in the context of approved standard treatments. The model can be used to assess whether the new therapy should replace the standard treatment, be combined with it or be used sequentially.

**Likely combination therapies**

The functional redundancy of the metastatic process ultimately mandates that drug combinations be developed.

**Oral or intravenous dosing**

When long-term drug administration is required, as in most metastasis prevention studies, oral dosing is imperative unless agents with long half-lives, such as monoclonal antibodies, are under development. Other routes of administration should be considered only if they are used clinically for the cancer type under study.

**Pharmacokinetics**

Collaborate with a pharmacologist, collect serum samples and generate pharmacokinetic data.

**Other end points**

Whenever possible, use end points in animal studies that closely reflect clinically relevant end points, such as disease-free survival, quality of life and overall survival, rather than tumour growth curves alone.

### Preclinical outcome measures

Development of metastases in preclinical models can be monitored by non-invasive imaging methods (for example, bioluminescence or MRI), enabling kinetic studies of their development. Standard metastasis models use imaging, histological counts and/or a quantitative measure of metastatic burden^45^ as primary end points, with survival as a secondary end point. Less often, other potentially important end points, such as rates of cell proliferation and apoptosis, microenvironmental alterations or immune infiltration are reported. This approach enables investigation of novel therapies but also the development of drug resistance that is associated with metastasis. Chemoresistance is often considered to be caused by gene mutations that negate the cytotoxic effects of therapy. Surprisingly, diverse metastasis pathways have been functionally implicated in chemoresistance in mouse models (Supplementary Box S1). Mechanistically, reductions in chemosensitivity have often been associated with activation of metastatic pathways that provide survival cues to the tumour cells, enhance proliferative signalling or counter DNA damage^84–86^. These findings uncover an unexpected intersection between metastasis and drug resistance that could lead to rational combinations of anti-metastatic agents and chemotherapies or other cytotoxic agents in clinical trials. These studies offer a provocative translational hypothesis, although careful studies using clinically achievable drug doses, schedules and combinations are needed in multiple preclinical model systems, including both chemotherapy-naive and chemoresistant models.

Another consideration when developing anti-metastatic therapies is whether to target tumour cells directly or indirectly by modifying the tumour microenvironment to be more suppressive to tumour growth. A common argument for targeting host cells is their greater genomic stability, which might make the development of drug resistance mechanisms less likely. However, the tumour microenvironment is complex and differs between each organ in which metastases have been established. Although not always adequate to predict metastatic events, preclinical models can help to understand the different host microenvironments and can be used to test therapies focused on site-specific metastasis. The unique microenvironment of bone provides the best example, relating to the aforementioned application of bisphosphonates and denosumab. In preclinical models of breast cancer bone metastasis, responses to bisphosphonates were found to be dependent on menopausal status: ovariectomized mice (mimicking a postmenopausal state) had a greater tumour burden in bone than control mice (mimicking a premenopausal state) but responded well to the therapy, whereas tumour burden in control mice was unaffected^87^. Likewise, in patients with breast cancer participating in the AZURE trial (zoledronic acid in combination with standard adjuvant therapy), a significant improvement in DFS (HR 0.75, 95% CI 0.59–0.96; *P* = 0.02) and OS (HR = 0.74, 95% CI 0.55–0.98; *P* = 0.04) was obtained only in women who had been postmenopausal for >5 years at the commencement of the trial^88^.

Development of pharmacodynamic markers indicative of drug activity must also be incorporated into preclinical testing. In clinical trials, traditional end points of radiological tumour responses and survival are increasingly inadequate. Thus, knowledge of whether the drug hits its intended target and how the tumour cells and/or tumour microenvironment respond is of growing importance. When designing experiments in animals, adequate consideration should be given to analyses that are achievable in patients — that is, liquid and/or tumour biopsy-based assessments and imaging. Circulating cell-free tumour DNA (ctDNA), tumour-derived exosomes or CTCs are increasingly common sources of pharmacodynamic markers that can be tested in mice and patients^89–94^.

### Comparisons with human tissues

Evidence of the activity of a prospective target using preclinical models is necessary but is not sufficient: assessment of relevance using clinical samples is imperative. Clinical evidence can be obtained retrospectively by analysing publicly available databases for the transcript of interest and assessing its prognostic and/or predictive value in a specified cohort of patients with cancer that has appropriate follow-up data. Such databases could also be used to identify potential predictive biomarkers for later study in clinical trials. Supplementing analyses with additional information, such as data on inactivation or mutations in genes specifically associated with metastasis, could also help build evidence for a particular target. If adopted, this approach must be used with caution, given that genetic alterations recorded in large databases might provide less robust targets for anti-metastatic agents than for other anticancer therapies because many metastasis pathways involve alterations in gene expression rather than mutations^95^. Any correlations must be confirmed at the protein level in tumour samples from a large number of patients, typically using tissue microarrays. For example, in preclinical models of spontaneous mammary tumour metastasis to bone, restoration of interferon regulatory factor 7 (IRF7) expression in the tumour cells did not alter primary tumour growth but did inhibit bone metastasis^44^. In a retrospective analysis of data from 855 samples from primary breast cancers, high expression of an IRF7 pathway gene signature was associated with reduced bone metastasis of breast cancer (HR 0.63, 95% CI 0.42–0.93; *P* = 0.021) but had no prognostic value in predicting metastasis to visceral organs^44^. In tissue array analyses, IRF7 protein was detected in 56% of primary tumours compared with 17% of distant metastases and in only 11% of bone metastases^44^. Prospective analyses can provide very strong evidence for potential therapeutic targets or biomarkers, but in the absence of the relevant cohorts for prospective analysis, appropriately designed prospective retrospective analyses can also generate good levels of evidence^96^.

Associations with non-coding RNA species can be assessed similarly through in situ hybridization in tissue samples. Metastatic biopsy samples are rarer than primary tumour specimens but are important for analyses of biomarker expression in the development of anti-metastatic therapies. Data must be interpreted with consideration of the patient’s prior treatments (or lack thereof) in terms of consistency with the proposed clinical setting in which the therapy will be developed.

### Drug properties

The desired pharmacological profile used to guide drug screening necessitates a shift from that typically accepted in an acute tumour shrinkage model to one of chronic, ideally oral administration — with the exception of a monoclonal antibody, for which monthly infusions might be considered feasible — with an appropriate risk–benefit profile. Optimization of structure–activity relationships can be used to select lead drug candidates with a pharmacokinetic profile appropriate for chronic use. For example, drugs that have good oral absorption characteristics and effective distribution to target tissues and are poor substrates for metabolic (cytochrome P450) enzymes in the liver (to avoid potential drug–drug interactions) are suited to simple once daily oral administration over months or years.

In the cancer setting, specific characteristics of the target disease, such as the frequency of metastasis to the brain, must also be considered^97–99^. For the example of brain metastasis, drug properties that increase blood–brain barrier permeability and reduce the potential for membrane efflux transporter elimination might be a priority. Toxicological structure alerts (toxicophores) need to be avoided in the development of any drug that is to be dosed chronically, particularly in elderly or frail patients who are likely to present with comorbidities, including reduced renal and liver function, or in patients who have previously been treated with multiple lines of anticancer therapy and are therefore likely to have a poor performance status. Avoidance of drugs that have high risk of hERG voltage-gated potassium channel blockade, which is associated with potentially fatal cardiotoxicity, is a special case in point^100^. Other pharmacological features associated with on-target and off-target toxicities also need to be avoided, particularly the generation of reactive metabolites that can cause carcinogenicity and nonspecific cytotoxicity.

## Clinical drug development

### Overview

Designing clinical trials and identifying end points that reflect the prevention of metastatic disease and can meet regulatory authority expectations for evidence of clinically significant benefit are arguably the most critical barrier to the development of new anti-metastatic agents. Measuring a clinically meaningful outcome in a realistic time frame, without requiring the recruitment of overwhelming patient numbers, is a key aim. The paucity of putative anti-metastatic agents being tested in clinical trials bears testament to these barriers.

Chemotherapies, hormone therapies, molecularly targeted agents, immunotherapies and various combinations thereof have produced responses and prolonged progression-free survival (PFS) and OS in the metastatic setting but are insufficient to achieve cure in most patients. Some of these same treatments have prevented or delayed the development of overt metastatic disease in some (but not all) patients when administered to those without detectable metastases but in whom micrometastases are suspected. Examples include the use of combination chemotherapy in the perioperative setting for bladder cancer^101,102^ and adjuvant tamoxifen for breast cancer^103^. This scenario has led to a drug development pathway in which therapies likely to be effective in eliminating micrometastases are progressed to the adjuvant setting only after they first demonstrate antitumour effects in patients with advanced-stage disease. Such an approach also assumes that the biology of the metastases is similar to that of the primary tumour, which is not universally true. This drug development paradigm is further reinforced by the need to manage the very large risks associated with trials in the adjuvant setting (for example, relating to the need for large cohorts, healthier populations and long durations for data maturity; the high costs; and the lack of safety data in patients with anticipated long-term survival) by first demonstrating activity and acceptable toxicity in trials involving smaller numbers of patients with more rapidly attainable end points. In other words, demonstrated efficacy in patients with pre-existing metastases has traditionally been the obligate gateway to adjuvant trials of interventions for metastasis prevention.

The ability to delay or prevent metastases has the potential to enormously improve the survival durations of patients with cancer and could even lead to cures. However, the opportunity presented by anti-metastatic drugs cannot be explored adequately using conventional drug development pathways because drugs without cytotoxic or clinically meaningful cytostatic effects in the patients with overt metastatic disease will never advance to adjuvant trials. This point is central to the rethinking of clinical trial designs for metastasis prevention, with the clinical use of potential anti-metastatic drugs falling into three possible scenarios.

#### Occult micrometastatic disease

A practical definition of micrometastatic disease is that which is suspected to be present at the time of treatment of the primary tumour but is not evident using conventional imaging and clinical examination. Micrometastases can sometimes be detected by other methods, such as in bone marrow aspirates using flow cytometry^104^, biochemical techniques (such as rising serum PSA levels in patients with prostate cancer^105^) or molecular assays for tumour-derived DNA (typically ctDNA in blood samples^106^). However, the predictive power of some of these tests is debatable, with questions remaining regarding their sensitivity and specificity^107^. Prospective randomized clinical studies in specific disease settings are required to examine the utility of these detection methods and thereby validate their use in guiding treatment decisions. Patients with micrometastasis can be identified across many, if not most, types of cancer, providing important new opportunities for anti-metastatic therapy. Occult micrometastatic disease might either be actively growing or dormant and how this difference might affect the choice and timing of therapy remains unclear. The detection of rising PSA levels in patients with no other evidence of disease after prior treatment of prostate cancer can be used as a platform for metastasis prevention (NCT03119857), although many trials simply use PSA metrics as a primary end point of treatment efficacy.

#### Tumours that cannot be removed surgically

In general, the aim of cancer surgery is to remove the primary tumour in order to obtain optimal local disease control and prevent the subsequent development of metastatic disease. Often, however, tumour resection is not feasible — despite the absence of metastatic disease — because of the anatomical location of the tumour or the likely inability of the individual patient to tolerate the proposed surgery (for example, owing to an insufficient respiratory reserve to endure pneumonectomy or insufficient fitness for prolonged anaesthesia). In practice, management of these patients presents a substantial clinical challenge. The use of drugs to prevent metastatic spread in addition to therapy to achieve local disease control (for example, by chemoradiation of locally advanced pancreatic cancer) is a potential approach to overcoming this challenge. This strategy could also be an alternative to tumour resection for patients in whom the morbidity and mortality associated with surgery would be better avoided. Another option for such patients arises from the emerging realization of the abscopal effects of radiotherapy — that local radiotherapy can lead to immune-mediated destruction of tumours outside the irradiated field^108^. Several clinical trials (NCT03323424, NCT03396471 and NCT02992912) are now testing the combination of local radiotherapy with immunotherapy to exacerbate the damage to overt metastatic lesions outside the radiation field^109,110^. If this hypothesis is proven, then the abscopal effect could potentially be harnessed for the management of micrometastatic disease to effect cures in future trials.

#### Patients at high risk of invasive malignancy

Metastasis is rare in the absence of an invasive primary tumour; thus, a strong clinical rationale exists for eliminating cancer in its pre-invasive state to prevent disseminated disease. Patients with discrete pre-invasive lesions might undergo major local therapy; for example, patients with non-invasive tumours of the bladder often undergo cystectomy^111^, and those with breast ductal carcinoma in situ can undergo mastectomy^112^. Even for some pre-cancerous conditions, the established organ-wide risk of pre-invasive disease (and thus malignant transformation) can warrant preventive surgery (such as pan-colectomy for familial polyposis) or regular surveillance with a view to major surgery (such as Barrett oesophagus). In some cases, complete removal of all at-risk tissue is not possible (for example, in patients with urothelial carcinoma in situ). Indeed, existing treatments are already used to reduce the risk of progression in patients with pre-invasive neoplasia (such as intra-vesical Bacillus Calmette–Guerin (BCG) immunotherapy for those with urothelial carcinoma in situ) or a known risk of developing cancer (for example, tamoxifen for women with an inherited risk of breast cancer owing to *BRCA* mutations). However, these approaches often have a disappointing effect on survival outcomes^113,114^, and the agents themselves might not target the pathways that most potently drive the invasive phenotype. Furthermore, continuous long-term administration of potentially toxic agents can be required to maintain the chemopreventive effect, which might not be feasible; for example, chemoprevention with retinoids in patients with pre-malignant head and neck tumours has been limited by poor tolerability (with the majority experiencing cheilitis, dry skin and conjunctivitis)^115^. Consequently, the opportunity to develop rationally targeted, more efficacious and better tolerated systemic therapies in such patient populations is attractive. In this scenario, anti-invasion therapies might be the most useful anti-metastatic approach.

### Challenges in clinical trial design

A dominant problem in each of the preceding scenarios is that intervention is likely to be required years in advance of the clinically important event (predominantly metastatic relapse or death). This latency presents substantial challenges for the design of statistically powered clinical trials that meet the regulatory standards for evidence. The main implications are both economic and clinical. Economically, the costs of trials with large cohorts and long follow-up durations are prohibitive, and the predicted return on investment is restricted by the potentially limited time remaining on a patent. Indeed, many current adjuvant therapies have come into routine use only after or near the time of patent expiry. For this reason, such studies are rarely industry funded. Clinically, the imperative is to make more rapid progress than this scenario allows.

The end points traditionally used in oncology clinical trials present an additional challenge. End points for determining antitumour efficacy in patients with advanced-stage disease continue to be based on conventional outcome measures including the objective response rate (ORR) based on reductions in tumour dimensions on cross-sectional imaging of established metastases, PFS and/or OS. Adjuvant trials typically have a primary end point of DFS. These indicators of efficacy either present a specific challenge to the timely development of anti-metastatic therapy or are not applicable in the absence of lesions that are detectable on imaging. Biomarkers that enable the detection of disease progression earlier than is possible through imaging of new metastatic lesions are clearly desirable; however, the use of such markers might require prospective validation, compounding the time and financial requirements.

With these challenges in mind, several ways forward are discernible. Owing to the plethora of potential drug candidates and the high costs of late-phase clinical studies, early clinical development must be maximized as follows. Anti-metastatic agents are unlikely to be substantially different from any other candidate anticancer drug in this regard, but early go versus no-go decisions will need to be based on robust mechanistic and other pharmacodynamic biomarkers rather than on the typical evidence of objective tumour responses in patients with advanced-stage disease. Given the essential favourable safety profile of these anti-metastatic drugs, efficient early phase clinical studies could feasibly be conducted in healthy volunteers; thus, the identification of biomarkers that are evaluable in non-malignant tissues will be of crucial importance.

The appropriateness of the novel end points will need to be supported by community-derived data providing compelling evidence that regulatory authority requirements will be met. By their nature, the novel end points are likely to be context-specific. Nevertheless, examples are available of accepted surrogate end points that have been used successfully in clinical development of drugs targeting pre-invasive pathways. These examples include the use of progression from early stage to stage T2 disease in establishing the role of intravesical BCG for the treatment of high-risk non-muscle-invasive bladder cancer^113^ and the use of a reduction in the numbers of polyps in the development of celecoxib for chemoprevention in patients with familial polyposis coli^116^. Early approval, on the basis on surrogate end points, will then need to be followed by confirmation of the clinical benefit in registry-type post-marketing studies.

In February 2018, the FDA also accepted the surrogate end point of metastasis-free survival in the first registration of apalutamide for the treatment of non-metastatic CRPC^117^. However, this end point was controversial^118^ because the use of conventional imaging (with isotope bone scans and CT) to establish the metastasis-free state will certainly result in failures to identify metastases that might be detectable using novel imaging modalities, such as whole-body MRI^119^ or ^68^Ga-prostate-specific membrane antigen-PET^120^. The use of these novel imaging techniques is unlikely to have changed the outcome of the trial (an imbalance between the arms in the incidence of MRI-detectable or PET-detectable metastatic disease seems unlikely); however, if patients had disease staging using these alternative, more sensitive imaging modalities, the control intervention (placebo alone) would have been inappropriate for the subset of patients with upstaging to metastatic disease, and thus the true therapeutic value of apalutamide might have been overstated. Hence, completion of dedicated studies to identify the true incidence of metastatic disease in particular patient populations might be necessary before such end points can be used to explore drugs with the specific aim of preventing metastasis.

Secondary prevention studies of metastasis are ongoing. In one such study (NCT03190967), patients with brain-metastatic breast cancer are being randomly assigned to receive metastasis prevention with the antibody–drug conjugate trastuzumab emtansine alone or in combination with metronomic temozolomide, with the primary objective of extending survival without new brain metastasis^121^. Similar end points are being explored in patients randomly assigned to a metastasis preventive strategy or control treatment after surgery for liver-limited metastatic colorectal cancer (for example, NCT03326791 and NCT00394992). Novel liquid biopsy assays of circulating tumour components, including ctDNA and CTCs, could also offer important opportunities for the development of biomarkers as new surrogate end points or intermediate markers to reduce the risk and accelerate the progress of anti-metastatic drug development (see the ‘Regulatory and registration pathways’ section)^91,122–126^.

Selecting populations at particularly high risk of having an early clinical event at a somewhat predictable rate for inclusion in first proof-of-concept trials — similar to the enrolment of patients at high risk of metastatic recurrence in the aforementioned studies of secondary metastasis prevention strategies — is a key approach to accelerating the development of anti-metastatic drugs. A wealth of literature is available describing prognostic factors that can help to identify such patients^127,128^; however, few studies have been completed prospectively, have incorporated independent validation cohorts or have used methodologies that can be accurately repeated and certified in diagnostic laboratories. The magnitude of metastatic risk is also an important consideration. Whether clinicians will feel comfortable designing a trial in a population with a 30% or 50% risk of metastasis over a defined period is debatable. The discovery of novel biomarkers that can be used to estimate the risk of micrometastases is a research area that has not been adequately addressed to date, although the use of ctDNA-based approaches to molecularly define minimal residual disease (MRD) might provide opportunities in this regard. Subgroups of patients with an especially high risk of early distant metastases are clearly identifiable. Examples include those with limited-stage small-cell lung carcinoma^129^, locally advanced pancreatic ductal adenocarcinoma^130^ or colorectal cancer and resectable liver metastases^131^. These disease settings are attractive both commercially and clinically, although reliance on such populations for early go versus no-go decisions holds the risk that effective anti-metastatic drugs will be prematurely discarded. Specifically, these high-risk populations might not display any degree of micrometastatic dormancy. Furthermore, these patients do not present an opportunity to explore drugs targeting pre-invasive targets.

Another way to improve the efficiency of late-stage drug development is to include only patients with disease in which the aberrant pathway being targeted is known to be active. This approach has other clear advantages to patients in that it avoids exposure to the toxicities of such drugs in those who are unlikely to benefit and enables their prioritization to receive other treatments. Indeed, this concept of precision medicine is rapidly becoming mainstream in all areas of anticancer drug development. To enable application of these principles to the development of anti-metastatic drugs, a full understanding of the link between the therapeutic target and the relevant subpopulation is vital, given that metastases are likely to have more complex and different drivers than those of the primary tumour. Heterogeneity between individual metastases adds an additional layer of complexity. Moreover, clinically applicable diagnostics should be developed in parallel with the drugs themselves. For some patients, however, time is of the essence in deciding on the next therapy; thus, a requirement to schedule biopsy sampling and evaluate the specimen in an accredited laboratory might present difficulties, particularly in the secondary metastatic setting, in which the oncologist and patient want to decide quickly on the next line of therapy (or trial participation). Conversely, in the postoperative adjuvant setting, this time delay might not be clinically relevant while the patient recovers from the primary surgical management. Similarly, when the aim is to evaluate the novel agent in a maintenance setting, the patient first needs to complete conventional postoperative systemic anticancer therapy.

Drugs that specifically target the processes of metastasis have the potential to transform the care of the majority of patients with solid tumours, although such agents are unlikely to be used as their sole therapy. One can envisage that treatment of the primary tumour and standard-of-care adjuvant therapy will remain the initial interventions, followed by potentially lifelong maintenance therapy to prevent the growth of pre-existing (micro)metastases and/or to prevent further spread of metastases. Thus, one challenging but necessary aim of clinical trials will be to establish the optimal combination and sequencing of these therapies with other modalities including surgery, radiotherapy, chemotherapy, molecularly targeted therapy and/or immunotherapy. The need to address the long-term toxicities of these drugs given the likely chronic duration of therapy is also essential.

## Regulatory and registration pathways

Many promising mechanistic biological targets exist for metastasis prevention strategies (Table 1); however, clear and feasible regulatory pathways for anti-metastatic agents are lacking. Specifically, the lack of surrogate end points appropriate for determining clinical efficacy at an early stage of drug development makes anti-metastatic agents unattractive to developers when compared with drug classes with rapid and proven pathways to registration.

A primary disincentive for anti-metastatic agents is the de facto use of time-to-event end points, such as PFS or OS, to assess efficacy in early stage clinical research. These end points present a far greater logistical and financial hurdle than the short-term ORR end points used to assess almost all newly registered oncology agents. In the period of 2006–2016, the FDA approved 180 oncology drugs (new and supplemental registrations), including 41 accelerated approvals: most accelerated approvals (*n = *37) were based on ORRs, with the remaining 4 approvals based on PFS using assessment criteria defined by indication-specific working groups^132^. The RECIST alone were used in the majority of studies that led to accelerated approvals. The RECIST and working group-derived criteria are standardized, well-defined and provide a rapid readout of objective tumour responses or disease progression^5^, whereas assessment criteria appropriate for anti-metastatic treatments are not standardized or clearly defined. These limitations are particularly problematic because the existing criteria designed for assessing objective responses or progression of solid tumours are unlikely to be of any use in assessing anti-metastatic agents that might have little effect on primary tumours; additionally, in most cases, the patient will not have any detectable disease at the time of treatment with such drugs.

In the absence of regulatory precedent or standardization, individual product developers will be required to explore new surrogate end points without certainty that regulators will accept their clinical relevance. Fortune rarely favours the brave in drug development. Even with a significant increase in biologically relevant bone-metastasis-free survival durations (4.2 month prolongation versus placebo, HR 0.85; *P* = 0.028) in 1,432 men with CRPC at high risk of developing bone metastases, the FDA denied an application for expansion of denosumab use to an anti-metastatic indication in this disease^133^. The regulator cited that it was “unclear whether an improvement in bone-metastasis-free survival alone in patients with CRPC at high risk of bone metastases is an adequate measure of clinical benefit in support of new labelling claims for a new patient population” (ODAC Briefing Document BLA 125320/28 Denosumab (XGEVA), 2012, FDA)^133^. This example underscores the risk involved in developing anti-metastatic agents and highlights the need to complete robust validation of putative new end points concurrently with the clinical development of such agents^134,135^.

Many other surrogate end points and biomarkers specific to metastatic progression have been considered, including the use of time-to-metastasis end points in patients at high risk of metastasis^121^, residual CTCs, ctDNA, DTCs and circulating tumour exosomes^91^. However, turning these end points and biomarkers into validated surrogates that regulators will endorse as the basis for accelerated approvals will require a collaborative approach to overcome the limitations of novel end points proposed by individual sponsors on an ad hoc basis^125^. These limitations include the high cost of biomarker development, the work required for refinement and standardization of test methods and the requirement for validation across comparable data sets and in sufficiently large numbers of the target population to support statistically robust conclusions^136^.

Plasma ctDNA profiling has demonstrated promise in detecting MRD and discriminating patients with and without eventual clinical recurrence following surgery and/or adjuvant therapy, notably in breast cancer^123,124,126,137^ and non-small-cell lung cancer^122^. These findings emphasize the potential use of ctDNA profiling to select patients at high risk of relapse following completion of adjuvant therapy for add-on anti-metastatic strategies, although these approaches remain in the research setting. Monitoring treatment response to immunotherapy using ctDNA-based liquid biopsy approaches might also be feasible^93^. Prospective, randomized trials are required to test whether persistent or rising ctDNA can be used as a surrogate for adjuvant therapy, alongside development of standardized workflows enabling clinical implementation. The implementation of such a strategy is illustrated by the accelerated FDA approval of blinatumomab in 2018 for the treatment of patients with B cell precursor acute lymphoblastic leukaemia who are in first or second complete remission but have an MRD burden ≥0.1%, which occurred on the basis of an end point relating to the achievement of undetectable MRD using a high-sensitivity detection assay after one cycle of treatment^138,139^. In addition, a Clinical Laboratory Improvement Amendments (CLIA)-registered duplex ctDNA blood test for colorectal cancer recurrence has been available in the USA since 2016 for surveillance of patients after tumour resection^140^.

The lack of fixed criteria for drug developers is an unacceptable disincentive, the effects being sadly evident in the lack of any new chemical entity initially registered for the prevention of metastasis. We urge regulatory agencies to work with researchers, drug developers and statisticians to identify and define guidelines for surrogate end points in order to encourage development of this under-represented category of oncology drugs.

Given that some anti-metastasis agents might be used in a preventive setting, the safety profile of such an Investigational Medicinal Product (IMP) will have to be much cleaner than those of standard anticancer agents. Proceeding to clinical trials even in the absence of adequate preclinical efficacy models might be acceptable, although a strong rationale will be needed to support long-term administration of the IMP in a healthier population of patients with cancer. The acceptable safety profile will be different for IMPs aimed at suppressing and/or eradicating established metastatic lesions. The benefit–risk assessment should include a detailed discussion of standard treatments, the temporal relationship with standard therapy (neoadjuvant, adjuvant or other applications), proposed safety monitoring and risk-minimization strategies; the potential for drug–drug interactions and risks associated with possible delaying of standard therapy should be addressed.

Development of new biomarkers as surrogate end points should be guided by correlations with previously validated end points and/or clinically relevant parameters (reviewed in ref.^141^). Companion diagnostics can be developed in parallel to the IMP and used for exploratory purposes. Once validated, the diagnostic can be used to dictate patient eligibility for trial participation and/or treatment assignment. In the USA, the FDA has provided guidance on validation of biomarkers for use in clinical development^142^, and in the European Union (EU), the European Medicines Agency has released guidance for biomarker analysis of clinical trial samples^143^. For marketing any test in the EU, Conformité Européene (CE) marking is required, indicating that the product meets EU safety, health or environmental requirements and compliance with EU legislation.

## Conclusions

Survival outcomes of patients with cancer have steadily improved since the advent of treatments including surgery, chemotherapy, radiotherapy, molecularly targeted therapies and immunotherapy. Nevertheless, in most patients who die of cancer, death is directly attributable to metastasis and not to the primary tumour. We have highlighted that development of effective therapies to treat and/or prevent metastatic disease requires a marked shift from the standard drug discovery and development paradigm.

**Fig. 3:**
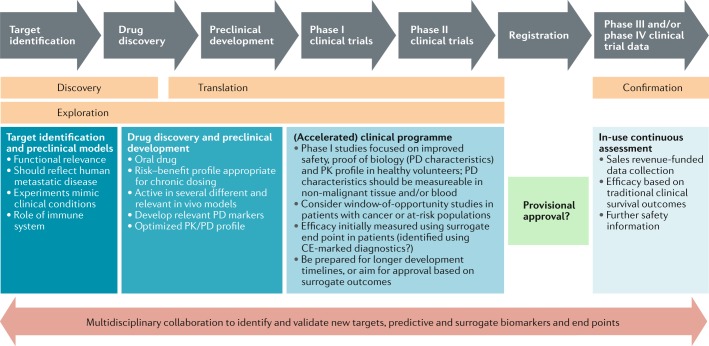
Development pathway for anti-metastatic agents. The general process for development of anti-metastatic agents has the same fundamental basis as that used in the development of drugs with a direct antitumour mechanism of action, with some special considerations as highlighted in the figure and described as follows. In target identification and preclinical development, special consideration must be given to the functional relevance of the models being used, which should reflect human metastatic disease as much as possible; the role of the immune system in metastasis is a critical factor. The experimental conditions should also mimic those of the clinical setting. Drug discovery and subsequent preclinical testing strategies need to be designed to account for the fact that, in most cases, the anti-metastatic therapy under development will be given chronically in a healthier population of patients, such as those that have been cured of their primary disease but are at high risk of developing secondary tumours, necessitating oral administration and a risk–benefit profile lacking key toxicity liabilities. Other considerations, such as activity in several different preclinical models, an optimized pharmacokinetic (PK) profile and development of pharmacodynamic (PD) markers suitable for use in the clinic, are common to all cancer drug discovery and development programmes. Given the favourable risk–benefit profile necessary for anti-metastatic agents, an accelerated development approach can be taken by conducting initial phase I studies in healthy volunteers rather than the classical populations with advanced-stage cancer. The key aims of these studies are to determine the safety, PK profile and PD characteristics (ensuring the putative biomarkers developed can be measured in non-malignant tissues) in order to provide an early go or no-go decision point and ensure that the drug has the intended biological effects. To gain rapid biological proof of concept in patients with cancer, window-of-opportunity studies, in which a dose of the anti-metastatic agent is given before surgery to examine PD effects, can be considered. If validated surrogate end points of clinical efficacy are available, these can be used to substantially reduce development timelines and, provided agreement has been reached with appropriate regulatory bodies, support provisional approval. If successfully executed, this regulatory strategy will avoid the protracted clinical development timelines that are one of the greatest barriers to the development of anti-metastatic drugs. Provided that provisional approval is given, regulatory bodies will require further in-use continuous assessment, typically in confirmatory phase IV studies that can be funded using ongoing sales revenue. The aim of these larger-cohort and much longer duration clinical trials is to confirm that a pre-defined level of clinical benefit is achieved according to more traditional outcomes, such as overall survival. If provisional approval has not been given by regulators, then costly (in terms of both finance and time) randomized controlled phase III studies in large cohorts will be necessary to gain approval on the basis of standard clinical outcome measures. CE, Conformité Européene.

Crucially, drug discovery programmes aimed at developing agents that specifically target metastasis should take into consideration the challenges and recommendations proposed herein; to facilitate such studies, we have provided summaries of our recommendations (Box 2) and overall development pathway (Fig. 3). Careful application of these lessons, learned from past failures, should maximize the probability of success in the development of this drug class. Regardless, continued discussion of these issues is warranted.

Indeed, considerable challenges remain, not least regarding the currently limited ability to detect pre-existing micrometastatic disease at first diagnosis. Establishment of new detection methods such as the Metas-Chip approach^144^, which uses a microelectronic biochip to detect micrometastasis using small-volume tumour and lymph node samples, will be needed. This method requires biopsy samples consisting of live cells and is based on the principle of detecting the migratory behaviour of tumour cells via their invasive capacity to retract single human umbilical vein endothelial cells from electrical sensing traps. Clear limitations of such assays include the logistical challenges associated with obtaining high-quality live biopsy material in the standard hospital setting and the high probability of no tumour cells being present in the tissue sample. The development of enhanced imaging techniques with greater resolution and sensitivity than those currently available, and their translation to widespread clinical application, will also be vital. Some examples under investigation include high-contrast fluorescence detection^145^, multispectral optoacoustic tomography^146^, shortwave infrared emitting nanoprobes^147^ and novel MRI contrast agents^148^. An ability to detect micrometastases, in addition to primary tumours and macrometastases, using these and other approaches will ensure that patients can be enrolled in clinical trials and assigned to appropriate treatment regimens.

Given the inherent additional complexity, timelines and potential cost of developing anti-metastatic agents, the support of regulators and the pharmaceutical industry will be crucial for future success. If this support can be provided, the potential improvements in the welfare of patients with cancer cannot be understated.

Box 2 Summary of recommendations for the development of anti-metastatic therapies **Target identification and preclinical models**

- Clear evidence and understanding of the functional relevance and biological activity of the proposed target in metastasis in humans are essential.
- Preclinical models must reflect the disease pathogenesis in patients; for example, the sites of metastasis should encompass the same or similar organs, and the target (or equivalent target in the model) must have the same role as in the human setting. Multiple models should be used.
- Experimental conditions should be designed to reflect the standard of care in the clinical setting and the desired route of treatment administration.
- Careful consideration should be given to the involvement of the immune system and role of chemoresistance within the models used.
  **Drug discovery and preclinical development**
- Anti-metastatic drugs are likely to be given via the oral route repeatedly over a prolonged period of time (perhaps years), necessitating a shift from the risk–benefit profile of traditional anticancer agents towards one with a considerably lower level of risk; the absorption, distribution, metabolism and excretion (ADME) profile of the drug should reflect this necessity.
- Drugs that have liver metabolism liabilities and/or common drug–drug interactions, undergo enzymatic hydrolysis or are substrates of membrane efflux transporters should be avoided.
- The pharmacokinetic parameters of the drug must be optimized to enable high levels of target exposure, including a high dissolution rate and good solubility and permeability.
- Lead clinical drug candidates should be tested in several different and, ideally, genomically complex models to account for diversity of the disease in patients.
- Pharmacodynamic markers need to be developed that reflect drug activity and can be translated to the clinical setting.
- Toxicology readouts should generally be cleaner than that often accepted for anticancer agents; for example, the drug should not result in blockade of hERG voltage-gated potassium channels nor generate reactive metabolites that can lead to downstream carcinogenicity or nonspecific toxicity.
  **Clinical development**
- Given the potential ethical issues surrounding the testing of experimental agents with an anti-metastatic mechanism, specifically relating to the very limited possibility of clinical benefit in patients with advanced-stage cancer, studies involving healthy volunteers should be considered as a first step in clinical testing.
- Early clinical development (ideally phase I expansion cohorts) should be focused on demonstrating proof of biological concept using validated pharmacodynamic markers relevant to the target and drug being tested; if healthy volunteers are enrolled in clinical trials, these markers must be measurable in non-malignant tissues and/or in blood.
- Any companion diagnostics that might have to be developed in parallel to the experimental therapeutic and Conformité Européene (CE) marked (indicating a product that meets European Union (EU) safety, health or environmental requirements and complies with EU legislation) can be used to guide treatment and/or trial enrolment decisions.
- Depending on the mechanism of action of the agent being tested, window of opportunity studies could also be considered during early phase clinical development to enable direct assessment of the biological mechanism in established primary or metastatic tumours.
- Alternate surrogate measures of clinical benefit, beyond traditional radiological criteria based on tumour shrinkage, are needed and will be dependent on the disease and setting being investigated; potential examples include end points based on time to appearance of new lesions and/or secondary lesions or levels of circulating tumour cells or cell-free tumour DNA.
- Initial proof-of-concept clinical studies can be conducted in selected patient populations at high risk of an early clinical event, such as a new metastasis. Thus, before and during clinical development, expert advice from oncologists with experience working with such patient groups should be sought. Moreover, the aberrant pathway under assessment should be confirmed as being active in this population.
- In light of the points above, clinical development timelines longer than those associated with traditional anticancer drugs should be anticipated; early approval based on surrogate outcomes should be a key goal, when possible.
  **Regulatory pathways**
- Ensure that a strong rationale is provided in regulatory submissions to support the long-term administration of the anti-metastatic agent in a population of healthier patients with cancer.
- Given the current absence of regulatory precedent or standardization, exploration of new surrogate end points should be discussed with the appropriate regulator before embarking on and during clinical drug development.
- If approval is given on the basis of short-term surrogate end points, a requirement to complete ‘sales revenue-funded’ phase IV confirmatory studies with larger cohorts, longer follow-up durations and more traditional end points, such as overall survival, should be anticipated.
- We urge regulatory agencies to work together with researchers, drug developers and statisticians to define guidelines on surrogate end points to encourage development of this complex but high-potential category of oncology drugs.

## Supplementary Information

Supplementary Box S1
