# Supplementary material for: A framework for the development of effective anti-metastatic agents
Source: Nat Rev Clin Oncol. 2018 Dec 4;16(3):185–204. doi: 10.1038/s41571-018-0134-8 (PMC7136167; doi:10.1038/s41571-018-0134-8)
Supplement: Supplementary file 1 — Supplementary Box S1 [file 41571_2018_134_MOESM1_ESM.pdf]

nature REVIEWS CLINICAL ONCOLOGY

SUPPLEMENTARY INFORMATION
In format as provided by the authors

A framework for the development of effective anti-metastatic agents

Robin L. Anderson, Theo Balasas, Juliana Callaghan, R. Charles Coombes, Jeff Evans, Jacqueline A. Hall, Sally Kinrade, David Jones, Paul S. Jones, Rob Jones, John F. Marshall, Maria Beatrice Panico, Jacqui A. Shaw, Patricia S. Steeg, Mark Sullivan, Warwick Tong, Andrew D. Westwell and James W. A. Ritchie, on behalf of the Cancer Research UK and Cancer Therapeutics CRC Australia Metastasis Working Group

https://doi.org/10.1038/s41571-018-0134-8

Supplementary Box S1 | **An unexpected functional intersection of metastasis and chemoresistance**

Mechanistic pathways in metastasis also modulate chemosensitivity in preclinical models. The highest level of evidence of this relationship emanates from preclinical models in which modulation of a specific signalling pathway altered both metastasis and chemosensitivity in vivo. Examples are as follows.

- The CXCL1/2–CXCR2 chemokine signalling pathway promotes metastasis of breast cancer by attracting myeloid cells that produce survival factors; inhibition of CXCR2 improved chemotherapeutic efficacy[S1].
- *BRAF* mutation combined with *PTEN* silencing accelerates melanoma metastasis[S2]. Integrin-mediated interactions of melanoma cells with activated fibroblasts in the tumour microenvironment increases ERK phosphorylation in the tumour cells, thereby augmenting resistance to BRAF inhibition[S3].
- Metadherin (MDTH) promotes metastasis by increasing adherence of tumour cells to endothelium[S4]. A *MDTH* DNA vaccine induces T cell responses against this cell adhesion molecule and sensitizes breast cancer to doxorubicin[S5].
- Overexpression of nucleoside diphosphate kinase A (NDKA) inhibits tumour cell viability during early lung colonization and metastasis[S6]. NDKA-overexpressing melanoma cells are more sensitive to cisplatin treatment, accumulating more DNA damage[S7].
- Overexpression N-cadherin increases the muscle invasion of prostate cancer; treatment with an anti-N-cadherin antibody delays progression of castration-resistant prostate cancer[S8].
- Knockdown of caveolin-1 reduces lung metastasis of renal cancer and enhances the efficacy of doxorubicin[S9].

These findings raise the possibility that interruption of the metastatic pathway will enhance the efficacy of chemotherapy, potentially providing opportunities for new combination clinical trial designs using existing standard of care treatments.

S1 Acharyya, S. et al. A CXCL1 Paracrine network links cancer chemoresistance and metastasis. *Cell* **150**, 165–178 (2012).

S2 Dankort, D. et al. Braf(V600E) cooperates with Pten loss to induce metastatic melanoma. *Nat. Genet.* **41**, 544–552 (2009).

S3 Hirata, E. et al. Intravital imaging reveals how BRAF inhibition generates drug-tolerant microenvironments with high integrin beta 1/FAK signaling. *Cancer Cell* **27**, 574–588 (2015).

S4 Hu, G. H. et al. MTDH Activation by 8q22 genomic gain promotes chemoresistance and metastasis of poor-prognosis breast cancer. *Cancer Cell* **15**, 9–20 (2009).

S5 Qian, B. J. et al. MTDH/AEG-1-based DNA vaccine suppresses lung metastasis and enhances chemosensitivity to doxorubicin in breast cancer. *Cancer Immunol.Immunother.* **60**, 883–893 (2011).

S6 Horak, C. E. et al. Nm23-H1 suppresses metastasis by inhibiting expression of the lysophosphatidic acid receptor EDG2. *Cancer Res.* **67**, 11751–11759 (2007).

S7 Ferguson, A. W. et al. Increased sensitivity to cisplatin by nm23-transfected tumor cell lines. *Cancer Res.* **56**, 2931–2935 (1996).

S8 Tanaka, H. et al. Monoclonal antibody targeting of N-cadherin inhibits prostate cancer growth, metastasis and castration resistance. *Nat. Med.* **16**, 1414–1496 (2010).

S9 Park, J. et al. RNA interference-directed caveolin-1 knockdown sensitizes SN12CPM6 cells to doxorubicin-induced apoptosis and reduces lung metastasis. *Tumour Biol.***31**, 643–650 (2010).
